# STROBE-GnRHa pretreatment in frozen-embryo transfer cycles improves clinical outcomes for patients with persistent thin endometrium: A case-control study

**DOI:** 10.1097/MD.0000000000029928

**Published:** 2022-08-05

**Authors:** Yixuan Liu, Lijuan Ma, Min Zhu, Huirong Yin, Hongli Yan, Minfeng Shi

**Affiliations:** a Reproductive Medicine Center, Changhai Hospital, Second Military Medical University, Shanghai, People’s Republic of China; b Department of Gynecology and Gynecological Oncology, Inselspital, Bern University Hospital, University of Bern, Bern, Switzerland; c Department for BioMedical Research, University of Bern, Bern, Switzerland.

**Keywords:** endometrial thickness, frozen-thawed embryo transfer, gonadotrophin-releasing hormone agonist, thin endometrium

## Abstract

The well-prepared endometrium with appropriate thickness plays a critical role in successful embryo implantation. The thin endometrium is the main factor of frozen-embryo transfer (FET), resulting in the failure of implantation undergoing FET. Hormone treatment is suggested to improve endometrium thickness; however, among the larger numbers of cases, it cannot reach the sufficient thickness, which leads to a high cancelation rate of embryo transfer as well as waste high-quality embryos. Thus, it increases the burden to patients in both economic and psychological perspectives. We performed a retrospective observational study, which was composed with 2 cohorts, either with the conventional hormone replacement therapy (HRT) protocol or HRT with gonadotrophin-releasing hormone agonist (GnRHa) pretreatment to prepare the endometrium before FET. The measurements of endometrium thickness, hormone level, transfer cycle cancelation rate, pregnancy rate, and implantation rate were retrieved from the medical records during the routine clinic visits until 1 month after embryo transfer. The comparisons between 2 cohorts were performed by t-test or Mann–Whitney *U* test depending on the different attributions of data. In total, 49 cycles were under HRT with GnRHa pretreatment and 84 cycles were under the conventional HRT protocol. HRT with GnRHa pretreatment group improved the endometrial thickness (8.13 ± 1.79 vs 7.51 ± 1.45, *P* = .031), decreased the transfer cancelation rate (*P* = .003), and increased clinical pregnancy rate and implantation rate significantly (both *P* = .001). Additionally, luteinizing hormone level in pretreatment group was consistently lower than conventional HRT group (*P* < .05). Our study revealed HRT with GnRHa pretreatment efficiently improved the endometrial thickness, therefore, decreased the FET cycle cancelation. It also elevated the embryo implantation rate and clinical pregnancy rate by improving endometrial receptivity.

## 1. Introduction

The frozen-embryo transfer (FET) has been largely promoted and accounted for 26% of all in vitro fertilization (IVF) cycles.^[1]^ The main impact factors of FET are embryo quality, number of transferred embryos and endometrial receptivity.^[2]^ The endometrial thickness is related to endometrial receptivity as the most important factors, and could be a predictor of success in IVF.^[3]^

It has been reported that the probability of nature clinical pregnancy for patient with thin endometrium was significantly low.^[4]^ As known, the thin endometrium is a critical factor in embryo implantation failure, impacting pregnancy outcomes after embryo transfer,^[5]^ increasing the risk of embryo transfer cancelation, causing the waste of the top-quality embryos and bringing the economic and psychological burden. There were around 25% of women aged 41 to 45 years and 5% with women aged younger than 40 years old suffering thin endometrium.^[6,7]^ The incidence of endometrial thickness < 7 mm on the day of HCG administration varies between 1% and 2.5% according to large-scaled prospective cohort studies among IVF populations.^[8,9]^ The incidence of thin endometrium is likely underestimated since these studies excluded patients with embryo transfer cancelation due to different reasons but mainly the thin endometrium.

Up to now, the definition of thin endometrium is still under debate. The sufficient thickness of endometrium is prerequisite for a successful implantation, which could be a certain lower limitations as 6 to 8 mm.^[5,10,11]^ Although 2 convincible studies applying the receiver operating characteristic curves, indicated the threshold of endometrial thickness for a possible pregnancy was above 8 mm.^[12,13]^ Thus, our study applied 8 mm as the cutoff value of thin endometrium.

The diverse etiology for thin endometrium mainly contains the repeated uterine curettage, chronic pelvic inflammation.^[14]^ Estrogen with sequential progesterone as hormone replacement treatment (HRT) is proposed as one of most popular treatments.^[15]^ However, the efficacy is limited even under combination therapy. Recent years, we modified the conventional HRT protocol, with the pretreatment of low-dose gonadotrophin-releasing hormone agonist (GnRHa). Reviewing dataset retrospectively, we investigated whether this optimized endometrial preparation protocol could improve the endometrial thickness and pregnancy outcomes, compared with conventional HRT.

## 2. Materials and Methods

As a retrospective study, all data were retrieved from electronic medical record system. We consecutively screened the patients with thin endometrium undergoing FET cycles in Reproductive Medicine Center of Changhai Hospital affiliated to Naval Medical University from January 2017 to January 2021. Our study was reviewed and approved by the local Ethical Review Board (201709471).

### 2.1. Selection of study population

All patients undergoing FET cycles were routinely measured the thickness of endometrium by ultrasound on the day of the ovum retrieval, or the day of progesterone administration, which was called the transformation day. Detailed measurements on endometrial thickness were described in following paragraph about measurement of endometrial thickness. Those patients with a 2-time <8 mm endometrium were screened into our analysis, defined as the cases with persistent thin endometrium.

All these patients underwent the endometrial preparation during FET cycles, either with the conventional HRT or HRT with GnRHa pretreatment. Hysteroscopy was routinely performed to confirm the integrity of endometrium. The cases with endometrium-related diseases including uterine malformations, uterine myoma, endometrial polyps, intrauterine adhesion, genital tuberculosis, and hydrosalpinx, were excluded from study cohort.

Hormone examinations were routinely requested during the day 2 to 5 of menstrual cycle, which was presented as baseline data, and repeated on transformation day.

### 2.2. Study cohorts and endometrial preparation protocols

Our study population were divided into 2 cohorts under the 2 different protocols for endometrium preparation during FET cycle. Part of patients repeated the same protocol and part of them switched to different protocols, such as from conventional HRT to HRT with GnRHa pretreatment.

**Figure 1.**
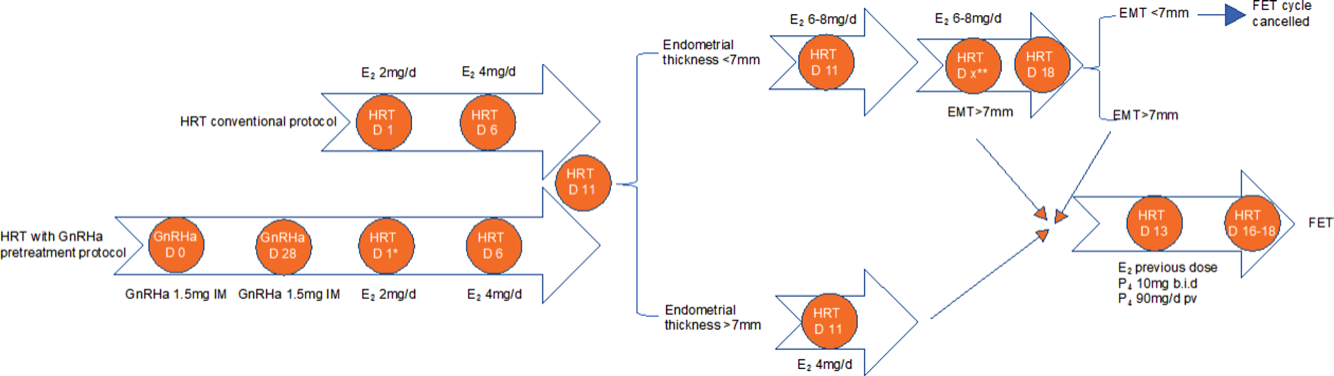
HRT protocols for endometrial preparation. It was usually started in menstrual bleeding day 3, which was presented as HRT D1 in HRT conventional protocol. *HRT D1 is on the 14th day after the second GnRHa injection. **HRT DX means any possible day between HRT D11 and D18. b.i.d. = 2-times per day, D = day, E_2_ = estrogen, orally administrated, FET = frozen-embryo transfer, HRT = hormone replacement therapy, IM = intramuscular, MC = menstrual cycle, P_4_ = progesterone, pv = per vagina.

The conventional HRT, for those regular-cycle patients, usually started on the day 3 of menstruation, namely HRT day 1 (HRT D1, Fig. 1). And for those irregular bleeding patients, it started on the day of induced-menstrual bleeding. The low dose of oral estradiol valerate (E_2_, Progynova; Bayer) 2 mg per day was last for 5 days, followed with gradual increased dose, according to the monitor of the endometrium thickness by transvaginal ultrasonography. The high dose of estradiol was normally last for another 2 days until that the endometrial thickness reached 7 mm. Otherwise, endometrium preparation would be evaluated on HRT day 18 (HRT D18, Fig. 1), which was HRT day 18, when the serum levels of LH, estrogen, and progesterone were examined. In condition that the endometrial thickness was <7 mm by HRT day 18 (HRT D18, Fig. 1) or that the progesterone level was higher than 1.5 ng/mL, this FET cycle was suggested to cancel. When endometrium reached 7 mm, progesterone was administrated 10 mg b.i.d and 90 mg of vaginal gel per day adjugated with E_2_ to mimic the menstrual cycles, which was transforming day. The detail process could refer to Figure 1.

In HRT with GnRHa pretreatment protocol, the long-term GnRHa, leuprorelin acetate microspheres were prescribed to patients with the sequential 2 doses. The first intramuscular injection of 1.5 mg GnRHa was administered on the day 2 to 5 of the menstrual bleeding. Another dose of 1.5 mg GnRHa was employed 28 days later. On the 14th day after the second injection, low dose of oral estradiol valerate was started as the same as described previously and followed progesterone when endometrium was prepared. Detail protocol was illustrated in Figure 1.

### 2.3. Measurement of endometrial thickness

**Figure 2.**
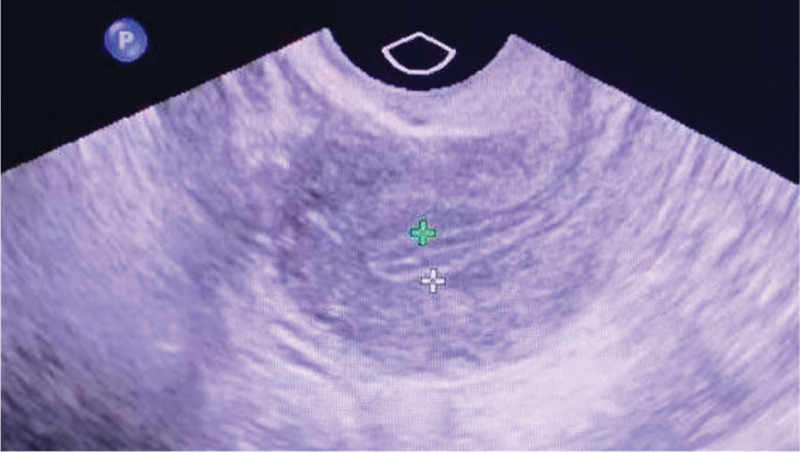
The measurements of endometrial thickness by transvaginal ultrasound. The uterus was in sagittal plane. The distance between 2 crosses as the thickest portion near the fundus was the endometrial thickness.

All measurements of endometrium were performed by transvaginal ultrasound using high frequency (38 MHz, model: 8; Philips), and under status of empty bladder. The thickness of endometrium was determined in the sagittal plane at the thickest portion near the fundus. The thickness was measured from 1 stratum basalis endometrial interface across the endometrial canal to the other stratum basalis interface (Fig. 2). The surrounding inner myometrial layer was excluded from the measurement. Endometrial thickness was measured routinely on the day of progesterone administration under these 2 endometrial preparation protocols.

### 2.4. Embryos transfer and supportive therapy in posttransfer

Embryos were assessed according to ASEBIR embryo evaluation criteria and Istanbul consensus.^[16]^ And the detail evaluation of top-quality embryos was also referring to the same recommendation.

FET was performed under the guidance of transabdominal ultrasound on the fourth or the sixth day after administration of progesterone.

Under progesterone prescription on the transforming day, high dose of 200 mg progesterone was suggested to taken orally every day from FET day onwards, until positive pregnancy test on 14th day after FET or clinical pregnancy. The serum human chorionic gonadotrophin (hCG) higher than 5 mIU/mL, was considered as the positive pregnancy test.

### 2.5. Follow-up on pregnancy outcome

The follow-up visits were conducted by nurses in a routine scheduled time frame after embryo transfer. Serum hCG and ultrasound examine were performed on day 14 and 30 to 35 after embryo transplant, respectively. Clinical pregnancy as an important outcome was confirmed by the increasing hCG and the presence of a gestational sac containing yolk sac at transabdominal ultrasonography. Ectopic pregnancy was not exclusive in this case. Implantation rate, a common definition referring to the previous report,^[17]^ was calculated by the percentage of the gestational sacs among the total embryos number transferred, which was obtained during routine follow-up.

### 2.6. Statistical analysis

Data analysis was done by the SPSS software (Statistical Package for the Social Sciences, version 18.0; IBM Corp, Armonk, NY). Continuous variables following normal distribution were given as mean ± standard deviation and compared by unpaired Student t-test, when following non-normal distribution were presented as median (interquartile range: P25–P75) and compared by Mann–Whitney *U* test. Categorical variables, presented as count and percentage, were analyzed by chi-square test or Fisher exact test. The statistical significance was assumed when 2-tailed value of *P* < .05. Multivariable logistic regression analysis was performed to identify potential factors contributing to clinical pregnancy for these special patients. Further subgroup analysis was also performed to limit the confounders.

## 3. Results

A total of 133 FET cycles from 76 patients were included into our analysis. Respectively, 49 cycles were under HRT with GnRHa pretreatment and 84 cycles were under the conventional HRT protocol.

### 3.1. Baseline characteristics

The baseline demographic and clinical variables from all included participants were shown in Table 1. The statistical comparison demonstrated no differences between 2 groups in terms of female age, male age, body mass index, duration of infertility, primary infertility rate, and the endometrial thickness on transformation day before HRT treatment. The serum sexual hormone levels were routinely examined during menstrual bleeding, presented in Table 1. And no differences were detected.

**Table 1:** Comparison of basic characteristics.

	GnRHa Pretreatment HRT (n = 49)	Conventional HRT (n = 84)	Statistical test	*P*
Female age (yr)	34.20 ± 5.37	34.46 ± 5.89	*t = *–0.254	.800
Male age (yr)	36.31 ± 7.28	36.39 ± 7.99	*t = *–0.062	.950
BMI (kg/m^2^)	22.07 ± 2.81	22.84 ± 3.96	*t* = 1.187	.238
Duration of infertility (yr)	4.0 (1.5–6.0)	3.0 (2.0–5.0)	*Z* = –1.055	.291
Primary infertility rate, n (%)	13 (26.5)	36 (42.9)	χ^2^ = 3.545	.060
b FSH (U/L)	6.20 ± 1.68	6.53 ± 2.38	*t* = 0.840	.403
b LH (U/L)	4.816 ± 2.47	4.490 ± 2.18	*t = *0.777	.439
b E_2_(pg/mL)	43.927 ± 28.23	37.42 ± 20.14	*t = *1.511	.133
b P (ng/mL)	0.56 ± 0.24	0.52 ± 0.23	*t* = –0.762	.448
Endometrial thickness before treatment (mm)	7.00 (6.25–7.50)	7.00 (6.03–7.50)	*Z* = –0.173	.863

### 3.2. Endometrial preparation outcomes

Table 2 displayed the detail about endometrial preparation outcomes. The endometrial thickness in HRT with GnRHa pretreatment group was significantly higher than that of conventional HRT group (8.13 ± 1.79 vs 7.51 ± 1.45, *P* = .031). The increased endometrial thickness (∆Em) after hormone treatments was significant larger in HRT with GnRHa pretreatment group compared with the conventional HRT group.

**Table 2:** Comparison of endometrial preparation outcomes on transformation day.

	GnRHa pretreatment HRT (n = 49)	Conventional HRT (n = 84)	Statistical test	*P*
Estrogen administration duration (d)	16.0 (15.0–17.0)	16.0 (14.0–17.0)	*Z* = –1.584	.113
LH (U/L)	0.86 ± 0.80	17.10 ± 11.43	*t = *–12.075	.000
E2 (pg/mL)	402.20 ± 556.83	460.88 ± 602.52	*t = *0.504	.615
P (ng/mL)	0.45 ± 0.27	2.29 ± 7.26	*t = *2.159	.034
Endometrial thickness (mm)	8.13 ± 1.79	7.51 ± 1.45	*t = *2.183	.031
∆Em (mm)	1.00 (0.15–2.50)	0.50 (0.00–1.40)	*Z* = –1.977	.048
Transfer cycle cancelation rate	6 (12.2)	30 (35.7)	χ^2^ = 8.635	.003

The actual duration of E_2_ administration and serum E_2_ level were similar between groups (*P* = .113, *P* = .615). However, significantly lower serum LH and progesterone level were observed as expected.

Thirty-six transfer cycle was canceled because of thin endometrium and/or high progesterone level on transformation day (*P* > 1.5 ng/mL). FET cycle cancelation rate was significantly lower in extended GnRHa plus HRT group than in conventional HRT group (12.2% vs 35.7%, *P* = .003, Table 2).

### 3.3. Embryo transfer and pregnancy outcomes

We analyzed embryo transfer and pregnancy outcomes in 43 and 54 transfer cycles among 2 groups respectively. Compared with conventional HRT group, there were significantly higher clinical pregnancy rate and implantation rate (53.5% vs 22.2%; 33.7% vs 14.0%; *P* = .001) in HRT with GnRHa pretreatment group (Table 3). The number of embryo transfer, top-quality embryo transferred per cycle and endometrial thickness on transfer day did not differ between the 2 groups (*P* > .05), and neither did the top-quality embryo transfer rate (52.3% vs 60.7%, *P* = .240). However, the blastocyst transfer rate in HRT with GnRHa pretreatment group was significantly higher than those in conventional HRT group.

**Table 3:** Comparison of embryo transfer and pregnancy outcomes.

	GnRHa pretreatment HRT (n = 43)	Conventional HRT (n = 54)	Statistical test	*P*
Endometrium thickness on transfer day (mm)	8.42 ± 1.61	8.15 ± 1.26	*t = *–0.969	.515
Number of embryos transferred per cycle	2 (2, 2)	2 (2, 2)	*Z* = –0.220	.826
LH level on transformation day (U/L)	0.87 ± 0.84	16.864 ± 10.92	*t = *10.419	.000
P level on transformation day (ng/mL)	0.46 ± 0.27	1.52 ± 2.51	*t* = 3.009	.004
Blastocyst transfer rate; n (%)	21 (48.8)	12 (22.2)	χ^2^ = 7.554	.006
Number of transferred top-quality embryo per cycle	1 (1, 2)	1 (0, 2)	*Z* = –1.024	.306
Top-quality embryo transfer rate; n (%)	45 (52.3)	65 (60.7)	χ^2^ = 1.380	.240
Clinical pregnancy rate; n (%)	23 (53.5)	12 (22.2)	χ^2^ = 10.147	.001
Ectopic pregnancy rate; n (%)	0 (0.0)	0 (0.0)	–	–
Implantation rate; n (%)	29 (33.7)	15 (14.0)	χ^2^ = 10.516	.001

### 3.4. Logistic regression analysis of factors associated with clinical pregnancy rate

For embryo transplant recipients, the factors that influence clinical pregnancy were analyzed using logistic regression (Table 4). Multivariable logistic regression analysis in transfer cycles showed that endometrium preparation protocol was associated with clinical pregnancy (*P* = .043). Instead, the odds ratio of conventional HRT for clinical pregnancy was 0.23 (95% confidence interval: 0.05–0.95).

**Table 4:** Logistic regression analysis of factors associated with clinical pregnancy rate.

	Multivariate analysis		
Variable	AdjOR	95% confidence interval	*P*
HRT protocols Conventional vs GnRHa pretreatment	0.23	0.05–0.95	.043
Female age (yrs)	0.93	0.85–1.02	.125
Number of transferred embryos	1.42	0.45–4.44	.548
LH level on transformation day (U/L)	1.01	0.95–1.07	.749
Blastocyst transferred, yes vs no	0.92	0.31–2.70	.872
Endometrium thickness on embryo transfer day (mm)	1.11	0.78–1.56	.565

### 3.5. Pregnancy outcomes in subgroups

To further analyze the effect of the 2 endometrial preparation protocols on pregnancy outcomes, we performed subgroup analysis among blastocyst transfer and the other embryo transfer. The results shown indicated that there was no difference on the endometrial thickness on transfer day, the number of transferred embryos and the E_2_ level on transformation day between HRT with GnRHa pretreatment and conventional HRT groups (*P* > .05). While the LH level on transformation day was much lower in HRT with GnRHa pretreatment group, in both subgroup analysis upon blastocyst transfer cycles or the other embryo transfer cycles (both *P* < .05). In subgroup analysis of blastocyst transfer cycles, although there was no significant difference in top-quality embryo, the clinical pregnancy rate and the implantation rate were significantly higher in HRT with GnRHa pretreatment group (both *P* < .05). Detail results were presented in Table 1 (Supplemental Digital Content, http://links.lww.com/MD/G951).

## 4. Discussion

Until now, the advantage of HRT with GnRHa pretreament remains controversial and there was no study to estimate its efficacy for patients with thin endometrium. Our study results showed that HRT with GnRHa pretreatment protocol with 2 continuous doses of pituitary suppression, might provide promising benefit to thin endometrium. This protocol significantly improved the endometrial thickness and decreased the cycle cancelation rate. There are similar results showing in the fresh embryo transplantation.^[18]^ To the best of our knowledge, this study is the first report on the clinical benefit of GnRHa pretreatment in thin endometrium patients with frozen-embryo transplantation.

The endometrium with certain thickness fosters embryo to attach, and provides nutrition for an implanting embryo during its first few weeks.^[19]^ Hence, the thickened endometrium is critical to the successful implantation and pregnancy. This study showed HRT with GnRHa pretreatment group improved the endometrial thickness significantly. And our analysis revealed that the FET cycle cancelation rate was significantly lower in HRT with GnRHa pretreatment group than conventional HRT group. This result was aligned with the case when GnRHa used in normal endometrial thickness. Prato et al^[20]^ reported a 4% cycle cancelation rate without GnRHa and 0% with 1 dose of GnRHa.

Further excluding the canceled cycles, there was no difference in endometrial thickness on transfer day between the 2 groups (Table 3), which was due to the artificial preference of cases with only thickened endometrium proceeds to the transplantation. Nevertheless, the pregnancy rate in the group of HRT with GnRHa pretreatment stayed higher. The latest report also showed clinical pregnant rate was increased from 37.68 to 46.1% among patients with various disease context under the GnRHa pretreatment.^[21]^ However, several previous studies showed conflicting results. Certain investigations reported similar pregnancy outcomes,^[22–25]^ while some indicated the superiority of HRT-FET with GnRHa^[21,26]^ or without GnRHa.^[27]^ But for certain infertility patients, GnRHa may be more beneficial. Many studies applied GnRHa pretreatment in patients with endometriosis or adenomyosis, which also revealed the better pregnant outcomes.^[28,29]^ Recent studies also have found that patients with unexplained repeated implantation failure, polycystic ovary syndrome, and high serum autoantibody levels could obtain better clinical outcomes with the GnRHa pretreatment.^[30–32]^ These above studies had the same trend of improvement on clinical outcomes with GnRHa pretreatment. However, due to the patient profile and GnRHa utilization in various studies vary largely, the direct comparison of the absolute number in detail is unreasonable. As to this study, we restricted to the infertile women with thin endometrium and the method of GnRHa use in this study was 2-time down-regulation.

We speculated that HRT with GnRHa pretreatment protocol could increase the clinical pregnancy rate not only by increasing the endometrial thickness but also by improving the endometrial receptivity. The underlying mechanisms could be that HRT with GnRHa pretreatment protocol improves endometrial receptivity via suppressing both the ovulation-induced LH surge and endogenous progesterone level. Furthermore, GnRHa acts directly on its local receptor in uterus. As previously reported, although the initiation of estradiol treatment at the early follicular phase in conventional HRT protocol could inhibit the ovulation,^[33]^ there were still certain spontaneous follicular activity,^[34]^ which might result in the LH surge and increase the endogenous progesterone level,^[35]^ thus impacted the endometrial receptivity. There are previous reports indicating that LH receptors have been localized in the endometrium.^[36,37]^ A recent study have reported that various regulatory alterations under surged LH were associated with the morphological and functional proliferation, as well as the differentiation of endometrial components, mainly via activation of the adenylate cyclase and phospholipase C pathways, accompany with increasing the local synthesis of steroid hormones.^[38]^ Our study identified the significant low levels of LH and progesterone on transformation day in HRT with GnRHa pretreatment group, which might result in the higher clinical pregnancy rate. In addition, relevant studies implied that the expression of the endometrial receptivity markers, such as LIF and integrin αvβ3, significantly increased in groups with the GnRHa pretreatment compared with the group without GnRHa.^[21]^ This study further revealed that GnRHa improved the endometrial receptivity via elevation of IL-6 and IL-11 in the endometrium. Meanwhile, in vitro experiments of endometrial stromal cells suggested that GnRHa regulated Il-6 and IL-11 through miR-124.

Although the transferred characteristics between study cohorts were similar, we noticed, in HRT with GnRHa pretreatment cohort, apart from the lower LH and endogenous progesterone level, there was a higher incidence of blastocyst transfer (48.8% vs 22.2%, *P* = .006). In the clinical practice of our clinical center, most of patients decided to have the blastocyst transfer when they underwent HRT with GnRHa pretreatment protocol in terms of time and economic concerns. As known, blastocyst transfer as the optimized embryo could improve the pregnancy rate. In order to adjust this bias, the subgroup analysis limited in blastocyst transfer cycles (refer to Table 1, Supplemental Digital Content, http://links.lww.com/MD/G951) showed the clinical pregnancy rate and implantation rate were significantly higher in HRT with GnRHa pretreatment group than those in conventional HRT group. Interestingly, among the subgroup of the nonblastocyst transfer cycles, 9 out of 22 cycles reached the clinical pregnancy in HRT with GnRHa pretreatment group, which was higher than 10 out of 42 cycles in conventional HRT group. But no statistical difference was reached potentially due to the small sample size.

As a retrospective study, the limitations of this study include its small sample capacity, unequal number of cases in 2 groups, and the inherent biases especially in blastocyst transfer. Hence, the logistic regression analysis and a subgroup analysis were performed to adjust this bias.

## 5. Conclusion

HRT with GnRHa pretreatment protocol, creating 2-time pituitary suppression before HRT, could lower the risk of cancelation of FET cycle via increasing the endometrial thickness, and improve the pregnancy related outcomes by optimizing the endometrial receptivity. The detailed underlying molecular mechanism could be very interesting to investigate in further researches.

## Acknowledgments

We thank the reproductive medicine center of Changhai hospital for offering the medical recording system to our study data retrieval.

## Author contributions

MFS and YXL designed this study and analyzed the data. HLY, MZ and HRY retrieved the data from medical recording system. YXL and LM conceived and revised this article. All authors contributed their input and agreed on article.

## Supplementary Material


