# Supplementary material for: STROBE-GnRHa pretreatment in frozen-embryo transfer cycles improves clinical outcomes for patients with persistent thin endometrium: A case-control study
Source: Medicine (Baltimore). 2022 Aug 5;101(31):e29928. doi: 10.1097/MD.0000000000029928 (PMC9351881; doi:10.1097/MD.0000000000029928)
Supplement: Supplementary file 1 [file medi-101-e29928-s001.pdf]

Supplementary Table 1 : Subgroup analysis of transferred characteristics and pregnancy outcomes

	GnRHa pretreatment HRT	Conventional HRT	Statistical test	P
**Non-blastocyst transfer cycles**				
Top-quality embryo transfer rate; n (%)	27 (60.0)	53 (63.1)	χ^2=0.119	0.730
Endometrial thickness on transfer day; (mm)	8.57±1.91	8.13±1.31	*T=-1.099*	0.276
Number of transferred embryos	2 (2,2)	2 (2,2)	Z=-0.420	0.675
P level on transformation day; (ng/ml)	0.39±0.23	1.73±2.82	T=2.946	0.005
LH level on transformation day; (U/L)	1.04±1.04	15.88±11.12	T=8.264	0.000
E_2 level on transformation day;(pg/ml)	523.17±688.67	431.21±592.82	T=-0.526	0.601
Clinical pregnancy rate; n (%)	9 (40.9)	10 (23.8)	χ^2=2.022	0.155
Ectopic pregnancy rate; n (%)	0 (0.0)	0 (0.0)	-	-
Implantation rate; n (%)	10 (22.2)	12 (14.3)	χ^2=1.305	0.253
**Blastocyst transfer cycles**				
Top-quality embryo transfer rate; n (%)	18 (43.9)	12 (52.2)	χ^2=0.405	0.525
Endometrial thickness on transfer day; (mm)	8.27±1.26	8.21±1.11	*T=-0.158*	0.876
P level on transformation day; (ng/ml)	0.536±0.30	0.843±0.64	*T=1.540*	0.145
LH level on transformation day; (U/L)	0.66±0.46	20.054±10.02	*T=6.698*	0.000
E2 level on transformation day; (pg/ml)	313.60±415.43	216.74±86.77	T=-0.907	0.377

Number of transferred embryos	2 (2,2)	2 (1.25, 2)	Z=-0.307	0.759
Clinical pregnancy rate; n(%)	14 (66.7)	2 (16.7)	χ^2=7.643	0.006
Ectopic pregnancy rate; n (%)	0 (0.0)	0 (0.0)	-	-
Implantation rate; n (%)	19（46.3）	3（13.0）	χ^2=7.242	0.007

LH: luteinizing hormone; E_2: estradiol; P: progesterone; transformation day is the starting day of progesterone adm
